# Differences in nurses’ perceptions of self-reported pain and the administered morphine dose according to the patient’s facial expression in Korea

**DOI:** 10.3352/jeehp.2020.17.38

**Published:** 2020-12-01

**Authors:** Jeong Yun Park, Da In Lee

**Affiliations:** 1 Department of Clinical Nursing, University of Ulsan, Seoul, Korea; 2 Department of Nursing, Seoil University, Seoul, Korea; Hallym University, Korea

**Keywords:** Cancer pain, Facial expression, Morphine, Pain management, Republic of Korea

## Abstract

**Purpose:**

This study aimed to compare nurses’ perceptions of self-reported pain, the recorded pain score, and pain treatment according to the patient’s facial expression.

**Methods:**

In this descriptive cross-sectional survey, the participants were 472 nurses working at a tertiary hospital in Seoul, Korea. A self-report questionnaire presented nurses with a smiling patient complaining of acute post-surgical pain and a grimacing patient with cancer pain, both of whom reported a pain level of 8 out of 10, and asked nurses to indicate their perception of the pain intensity, the pain score that they would record, and the medication that they would provide for each patient.

**Results:**

The pain intensity perceived by nurses for the grimacing patient was significantly higher than that for the smiling patient (P<0.001). The recorded pain score was likewise significantly higher for the grimacing patient than for the smiling patient (P<0.001). There was a significant difference in the amount of morphine chosen by the nurses for pain interventions between the smiling and grimacing patients (P=0.040). Higher perceived pain intensity and score were associated with higher administered doses of morphine.

**Conclusion:**

These findings suggest that nurses might be affected by patients’ facial expressions when treating pain. A pain management program should be developed that trains nurses to accurately recognize pain hidden in patients’ faces and provides them with the knowledge of how to appropriately assess and manage patients’ pain.

## Introduction

### Background/rationale

In 2004, the Korean Ministry of Health and Welfare developed and put into practice guidelines for cancer-pain management [1]. Since 2007, pain care has been included in the accreditation criteria of Korean medical institutions to determine whether they have an appropriate system for pain assessment and management. These guidelines and criteria have led to a greater awareness of pain among medical personnel. To further develop pain guidance that is appropriate for each medical institution, multidisciplinary workshops and educational programs that include instruction in pharmacological and nonpharmacologic pain relief therapies have been developed and implemented in Korea [2]. Multidisciplinary interventional programs were necessary because of differences between nurses and physicians in their knowledge and awareness of pain control for cancer patients [3].

In the United States, approximately 75% of post-surgical patients experienced moderate to severe pain [4], and more than 38.0% of cancer patients were found to suffer from severe pain [5]. Pain control is hindered by an inappropriate assessment of the level of pain, especially when patients are reluctant to express their symptoms and nurses cannot accurately assess pain levels [3]. Although most nurses accept the use of analgesics to control patient pain, they tend to use fewer analgesics for fear of side effects and addiction [6]. In Korea, it was found that 50% to 80% of cancer patients did not receive proper pain treatment due to passive attitudes toward pain and perceptions of pain management [7]. Furthermore, the World Health Organization rated Korea as being at a moderate level in the adequacy of opioid analgesic consumption, because the use of analgesics in Korea showed an adequacy of consumption measure (ACM) of 47.0%, compared to the country with the highest ACM value (Canada, 312.6%) [8].

Self-reported pain scores are critical indicators of pain management [9]. Nurses must trust these pain scores and decide upon an appropriate intervention accordingly. However, nurses sometimes differentiate the intensity of the pain reported by patients based on their facial expressions, and patients’ masking of pain may result in inadequate pain management [10]. The pain levels reported by nurses and patients are often different, but only limited research has investigated pain management provided by nurses based on patients’ behavior and facial expressions [4,6].

Nurses deal with various pain symptoms in clinical practice, including difficulties in communicating pain, acute pain in cancer patients, chronic pain, and complex pain complaints, and they are required to provide appropriate interventions. Nurses’ assessments of pain and interventions to treat pain play an essential role in effective pain control for patients [11]. However, previous studies have focused on the knowledge and attitudes of patients [5] and medical staff as factors affecting self-reporting and pain evaluation [7]. Insufficient case studies have investigated how evaluations of pain intensity and pain management may vary depending on patients’ facial expressions.

### Objectives

This study aimed to investigate and analyze the perceptions of pain intensity, recorded pain scores, and medication determined by nurses in 2 different situations involving pain patients and to provide insights into the development of practical pain-management education programs for implementation in clinical environments. The hypotheses of the study were as follows:

Hypothesis 1: Nurses will perceive the pain intensity as the pain rating given by the patient.
Hypothesis 2: Nurses will record the same pain intensity for both the smiling patient and the grimacing patient.
Hypothesis 3: Nurses will administer an appropriate dose of morphine to both the smiling paient and the grimacing patient equally.

## Methods

### Ethics statement

Approval from the Institutional Review Board of Asan Medical Center (approval no., 2013-0312) was obtained. Informed consent was obtained from the participants.

### Study design

This study administered a descriptive cross-sectional survey to nurses who worked at Asan Medical Center in Seoul, Korea.

### Setting

The data collection period was from April 22 to 29, 2013. A total of 486 responses to questionnaires were collected, of which 472 were analyzed after excluding questionnaires with insufficient answers. The raw data can be found in Dataset 1.

### Participants

A convenience sample of nurses in the nursing department of Asan Medical Center was recruited for this study. All nurses who were working in 23 clinical wards were given information about the study and asked to participate. To ensure a wide range of experience, newly-hired nurses were excluded. Only nurses who agreed to complete the survey were selected for the study. A total of 472 nurses completed the survey. The demographic characteristics of the nurses participating in this study are presented in Table 1.

### Data sources/measurements

The pain management assessment tools developed by McCaffery and Ferrell [12] were used with permission. This tool presents 2 patients with pain as clinical cases. The patients are identical except for their behavior. Case 1 is presented as a smiling patient complaining of acute post-surgical pain, whereas case 2 is presented as a grimacing patient with cancer pain. The self-reporting questionnaire requires participants to answer 3 questions for each case. For each patient, the participant is asked to identify their personal opinions about the patient’s pain intensity, what they will record in the patient’s records, and what opioid dose they will administer.

There is no correct answer to question 1 about the participant’s personal opinion. The correct answer for question 2 for each patient is to record an 8 (the pain rating given by the patient). For question 3, the correct answer is to administer to each patient 3 mg of morphine, a 50% increase from a previously safe but ineffective opioid dose (Supplement 1). An English translation of the clinical cases is available in Supplement 2.

The measurement tool was tested for validity and reliability. The questions were verified, corrected, and supplemented by consulting with 1 professor, 2 unit managers, and 2 clinical nurse specialists. The Cronbach α value was 0.769.

### Study size

A post hoc power calculation was conducted using G*Power ver. 3.1.9.4 (Heinrich-Heine-Universität Düsseldorf, Düsseldorf, Germany; http://www.gpower.hhu.de/). For the paired t-test, the input was as follows: tails=2, effect size (d)=0.5, alpha error probability=0.05, and total sample size=472. The power was calculated as 1.0.

### Quantitative variables

None.

### Statistical methods

Collected data were analyzed using IBM SPSS ver. 22.0 (IBM Corp., Armonk, NY, USA). Descriptive statistics were used to analyze demographic data. Frequencies and the independent t-test were used to compare responses to the 3 questions for each of the cases. The paired t-test was used to analyze differences in the nurses’ responses to the 3 questions between the 2 patients with different facial expressions. Correlations between the perceived pain intensity, recorded pain score, and pain intervention by nurses according to the patient’s facial expressions were analyzed.

## Results

### Comparison of nurses’ perceptions of pain intensity, recorded pain intensity, and pain scores between the smiling and grimacing patients

The pain intensities, as represented by pain scores, perceived by the nurses for the smiling and grimacing patients are presented in Table 2. The average pain intensity perceived by nurses was significantly higher (t=-12.37, P<0.001) for the grimacing patient (7.11±2.57) than for the smiling patient (5.86±2.60). The distribution of pain scores recorded by the nurses as part of the pain assessment is shown in Table 2. The pain score was significantly higher (t=-5.79, P<0.001) for the grimacing patient (7.97±1.93) than for the smiling patient (7.71±1.96).

### Morphine dose administered for pain management

Table 3 shows nurses’ responses regarding the morphine dose that they would administer for the pain management of the smiling and grimacing patients. There was a statistically significant difference (χ^2^=-2.75, P=0.040) in the amount of morphine chosen by the nurses for the pain intervention in the smiling and grimacing patients.

### Correlations between nurses’ perceptions of pain, recorded pain scores, and interventions in the smiling and grimacing patients

For the smiling patient, the perceived pain intensity was significantly correlated with the recorded pain score (r=0.58, P<0.001) and the pain intervention (r=0.29, P<0.001). The pain record was significantly correlated with pain intervention (r=0.28, P<0.001). For the grimacing patient, the perceived pain intensity was also significantly correlated with the recorded pain (r=0.58, P<0.001) and the pain intervention (r=0.15, P<0.001). Similarly, the recorded pain was significantly correlated with the pain intervention (r=0.21, P<0.001).

## Discussion

### Key results

This study investigated and analyzed how nurses perceived, recorded, and selected interventions for self-reported pain according to the patient’s facial expressions. The pain rating given by both the grimacing patient and the smiling patient was 8. The pain intensity perceived by the nurses was 7.11 points for the grimacing patient, which was significantly higher (t=-12.37, P<0.001) than the perception of 5.86 points for the smiling patient reporting the same pain level. The recorded pain was 7.71 points for the smiling patient and 7.97 points for the grimacing patient. The recorded pain for the grimacing patient was significantly higher to a remarkable extent (t=-5.79, P<0.001). Since patients reported their pain scores to be 8 points and the appropriate dose of morphine for this pain level is 3 mg, nurses perceived, recorded, and responded to the patient’s pain at lower levels than the actual pain intensity of the patient. These findings show that nurses may not record actual pain scores reported by patients or administer adequate doses of analgesics, even though they are required to do so.

### Interpretation

We need to acknowledge and limit the negative effects that subjectivity may have on patient care, but it is impossible to exclude subjective judgment completely. Nurses’ perceptions of pain intensity, the recorded pain, and pain intervention according to the patient’s facial expression showed significant correlations, but the variable most significantly impacting the pain intervention was perceived pain intensity. Perception is an important factor in assessing a patient’s pain. The pain intervention provided to a patient may vary depending on how a nurse perceives a patient’s pain intensity, rather than on how the patient communicates his or her pain. The above finding shows that a more intense pain intervention would be given to the grimacing patient than to the smiling patient, suggesting that a patient’s smile was interpreted by nurses as a lower pain level. A nurse needs to be able to identify hidden pain that is not evident in a patient’s facial expression, given that laughter may be an effective way to relieve pain.

### Comparison with previous studies

The McCaffery and Ferrell [12] vignettes were used to assess nurses’ knowledge and management of pain. Self-reported pain is the most reliable indicator of pain intensity. Pain knowledge involves understanding the subjective experience of pain, and thus, believing and acting on patients’ self-reports [10], despite objective manifestations. Another factor that caused the difference in pain care is that grimacing is the most reliable expression of pain in both verbally and cognitively impaired patients. However, people may smile, despite their pain, for reasons other than cognitive impairment. Nurses should judge carefully whether a patient’s words are reliable. Critical thinking competency in pain assessment is required for effective pain management. A gap exists between the pain intensity recorded in medical records and the pain intensity reported by patients [13]. Because nurses subjectively assess pain intensity according to patients’ facial expressions, subjective judgments must be excluded from the patient evaluation to ensure a correct pain intervention. In a study comparing pain intensity according to facial expression, it was found some patients tried to hide their pain even when it was severe. To provide appropriate interventions for these patients, it is necessary to educate nurses about pain assessment and intervention [7]. The factor most influencing inappropriate pain intervention in cancer patients was the difference between the pain intensity as assessed by medical staff and by the patients. Patients display different facial expressions and responses to the same pain intensity because of the variety of human coping mechanisms, and nurses should be aware of the fact that they might be influenced by patients’ facial expressions when treating pain [14]. A patient’s consciousness also is expressed in a specific context. In most clinical cases, patients are assessed on 1 dimension of pain, but it is important to assess both sensory perceptions and perceptions of unpleasantness [15].

### Limitation

This study only investigated nurses based on a presented case study. To translate these findings into actual patient care scenarios, further investigations should be repeated at multiple hospitals in real time, using video recordings or a series of videos.

### Generalizability

Even though the study subjects were all volunteers, the high response rate suggests that nurses in this hospital considered pain management to be a critical nursing issue. The results of this study may therefore be applied to nurses in Korea.

### Conclusion

The study revealed that nurses perceived lower-intensity pain than the pain rating given by the patient and nurses recorded a higher intensity of pain for the grimacing patient than for the smiling patient. Furthermore, this study confirmed that nurses administer inappropriate doses of morphine to patients with pain. Therefore, the first hypothesis was accepted; however, the second and third hypotheses could not be accepted. It is critical that nurses understand the variety of patients’ responses to pain in order to assess the patient's pain intensity clearly and provide appropriate pain interventions. Nursing standards could help nurses provide treatment based on a patient’s needs by excluding subjective judgment and recording the patient’s stated pain intensity. It is necessary for nurses to carefully understand a patient’s responses to pain, to assess the pain intensity clearly, and to provide the appropriate intervention. To provide nurses with knowledge on how to appropriately assess and manage patient pain, a pain-management program that enables nurses to accurately recognize pain hidden in patients’ faces should be developed. This will lead to changes in the awareness of pain management and improve the effectiveness of pain assessment, prevention, and interventions for patients.

## Figures and Tables

**Table 1.** Demographic characteristics of nurses (N=472)

Variable	Category	No. of participants (%)
Age (yr)	<30	371 (67.2)
	30–39	131 (27.8)
	≥40	24 (5.1)
Marital status	Single	363 (76.9)
	Married	109 (23.1)
Education	Diploma	46 (9.7)
	Baccalaureate	349 (73.9)
	≥Master’s degree	77 (16.3)
Clinical experience (yr)	<1	72 (15.3)
	1–3	106 (22.5)
	4–5	119 (25.2)
	6–10	105 (22.2)
	>10	70 (14.8)
Position (title)	Staff nurse	428 (90.7)
	Charge nurse	22 (4.7)
	Unit manager	15 (3.2)
	Clinical nurse specialist	7 (1.5)

**Table 2.** Nurses’ perceptions of pain intensity and recorded pain assessment (N=472)

Pain assessment scale	Perceived intensity			Recorded pain intensity		
	Smiling patient	Grimacing patient	t-value (P-value)	Smiling patient	Grimacing patient	t-value (P-value)
Pain score	5.86±2.60	7.11±2.57	-12.37 (<0.001)	7.71±1.96	7.97±1.93	-5.79 (<0.001)
0	8 (1.7)	2 (0.4)		7 (1.5)	3 (0.6)	
1	9 (1.9)	4 (0.8)		7 (1.5)	3 (0.6)	
2	91 (19.3)	7 (1.5)		54 (11.4)	9 (1.9)	
3	99 (21.0)	30 (6.4)		56 (11.9)	26 (5.5)	
4	56 (11.9)	47 (10.0)		35 (7.4)	37 (7.8)	
5	22 (4.7)	37 (7.8)		9 (1.9)	23 (4.9)	
6	11 (2.3)	39 (8.3)		7 (1.5)	26 (5.5)	
7	3 (0.6)	6 (1.3)		1 (0.2)	5 (1.1)	
8^a)^	173 (36.7)	294 (62.3)		296 (62.7)	331 (70.1)	
9	0	6 (1.3)		0	9 (1.9)	
10	0	0		0	0	

Values are presented as mean±standard deviation or number (%).

a) Correct answer.

**Table 3.** Morphine dose administered by nurses to alleviate the patient’s pain (N=472)

Pain intervention	Smiling patients	Grimacing patients	χ^2^ (P-value)
No morphine at this time	106 (22.5)	78 (16.5)	-2.75 (0.040)
Morphine 1 mg IV now	118 (25.0)	108 (22.9)	
Morphine 2 mg IV now	171 (36.2)	186 (39.4)	
Morphine 3 mg IV now^a)^	77 (16.3)	97 (20.6)	

Values are presented as number (%). IV, intravenous.

a) Correct answer.

## Footnotes

**Data availability** Data files are available from Harvard Dataverse: https://doi.org/10.7910/DVN/0SNQKN Dataset 1. Raw data of the response from 472 participants.

## Supplementary materials

Data files are available from Harvard Dataverse: https://doi.org/10.7910/DVN/0SNQKN

Supplement 1. Measurement tool in Korean.

Supplement 2. Measurement tool translated into English.

Supplement 3. Audio recording of the abstract.
