# Supplementary material for: Differences in nurses’ perceptions of self-reported pain and the administered morphine dose according to the patient’s facial expression in Korea
Source: J Educ Eval Health Prof. 2020 Dec 1;17:38. doi: 10.3352/jeehp.2020.17.38 (PMC7884257; doi:10.3352/jeehp.2020.17.38)
Supplement: Supplementary file 2 — Supplement 1. Measurement tool in Korean. [file jeehp-17-38-suppl1.doc]

**Supplement 1. 설문도구**

|  | | | | |
| --- | --- | --- | --- | --- |
| * 사례 1: **50세 남자 환자가 복부수술 후 1일째 되었다.** 간호사가 병실에 들어섰을 때 환자는 웃으면  서 방문객과 대화를 나누고 있었다. 혈력징후를 측정한 결과 혈압 120/80 mm Hg, 맥박 80회/분, 호흡  18회/분이었고, 통증강도를 환자에게 10점 숫자 척도(0=통증 전혀 없음, 10=통증 가장 심함) 측정했을  때 환자는 8점이라고 답하였다. | | | | |
| 1. 아래 통증 척도에 귀하의 생각에 일치하는 환자의 통증 강도를 동그라미표 해주십시오. | | | | |
|  | | 0 1 2 3 4 5 6 7 8 9 10 |  | |
| 통증 없음 통증 심함 | | | | |
| 2. 환자의 통증기록지에 기록해야 합니다. 귀하가 기록할 통증 강도를 동그라미표 해주십시오. | | | | |
|  | 0 1 2 3 4 5 6 7 8 9 10 | | |  |
| 통증 없음 통증 심함 | | | | |
| 3. 환자는 통증 호소로 2시간 전에 morphine 2 mg을 IV로 투여받았다. Morphine 투여 후 통증 정도를 사정한 결과 ‘주사를 맞고 나서 반시간 정도 6점에서 8점 범위라고 표현하였으며, 2점 이하로 내려가면 심호흡이나 기침을 하기가 편할 것 같다고 하였다. 환자는 호흡억제나 진정작용 등의 부작용은 없었다. PRN (as needed) 처방으로 mild pain이면 morphine IV 1 mg q1h (every hour), moderate pain이면 morphine IV 2 mg q1h, severe pain이면 morphine IV 3 mg q1h을 처방한 상태이다.  귀하께서는 이 상황에서 어떤 결정을 내리시겠습니까?  1) 지금은 morphine을 투여하지 않는다.  2) Morphine 1 mg을 IV 한다.  3) Morphine 2 mg을 IV 한다.  4) Morphine 3 mg을 IV 한다. | | | | |

| * 사례 2: **75세 여자 환자가 허리 통증으로 입원하였다**. 간호사가 병실에 들어섰을 때 환자가 침대에서  누어 있다가 자세를 바꿀 때 얼굴이 찌푸리는 것을 관찰할 수 있었다. 혈력징후를 측정한 결과 혈압  120/80 mm Hg, 맥박 80회/분, 호흡:18회/분이었고, 환자의 통증강도는 10점 숫자 척도(0=통증 전혀 없음,  10=통증 가장 심함) 측정했을 때 8점이라고 답하였다. | | | | |
| --- | --- | --- | --- | --- |
| 4. 아래 통증 척도에 귀하의 생각에 일치하는 환자의 통증 강도를 동그라미표 해주십시오. | | | | |
|  | | 0 1 2 3 4 5 6 7 8 9 10 |  | |
| 통증 없음 통증 심함 | | | | |
| 5. 환자의 통증기록지에 기록해야 합니다. 귀하가 기록할 통증 강도를 동그라미표 해주십시오. | | | | |
|  | 0 1 2 3 4 5 6 7 8 9 10 | | |  |
| 통증 없음 통증 심함 | | | | |
| 6. 환자는 통증 호소로 2시간 전에 morphine 2 mg을 IV로 투여받았다. Morphine 투여 후 통증 정도를 사정한 결과 ‘주사를 맞고 나서 반시간 정도 6점에서 8점 범위라고 표현하였으며, 2점 이하로 내려가면 심호흡이나 기침을 하기가 편할 것 같다고 하였다. 환자는 호흡억제나 진정작용 등의 부작용은 없었다. PRN 처방으로 mild pain이면 morphine IV 1 mg q1h, moderate pain이면 morphine IV 2 mg q1h, severe pain이면 morphine IV 3 mg q1h을 처방한 상태이다.  귀하께서는 이 상황에서 어떤 결정을 내리시겠습니까?  1) 지금은 morphine을 투여하지 않는다.  2) Morphine 1 mg을 IV 한다.  3) Morphine 2 mg을 IV 한다.  4) Morphine 3 mg을 IV 한다. | | | | |
