# Supplementary material for: Differences in nurses’ perceptions of self-reported pain and the administered morphine dose according to the patient’s facial expression in Korea
Source: J Educ Eval Health Prof. 2020 Dec 1;17:38. doi: 10.3352/jeehp.2020.17.38 (PMC7884257; doi:10.3352/jeehp.2020.17.38)
Supplement: Supplementary file 3 — Supplement 2. Measurement tool translated into English. [file jeehp-17-38-suppl2.doc]

**Supplement 2. Measurement tool**

|  | | | | |
| --- | --- | --- | --- | --- |
| * **Case 1**: A is 25 years old and this is his first day following abdominal surgery. As you enter his room, he smiles at you and continues talking and joking with his visitor. Your assessment reveals the following information: blood pressure (BP), 120/80 mm Hg; heart rate (HR), 80 beats/min; respiratory rate (R), 18 beats/min; on a scale of 0 to 10 (0, no pain/discomfort; 10, worst pain/discomfort) he rates his pain as 8. | | | | |
| 1. Circle the number that you rate the A’s pain. | | | | |
|  | | 0 1 2 3 4 5 6 7 8 9 10 |  | |
| No pain/discomfort Worst Pain/discomfort | | | | |
| 2. On the patient’s record you must mark his pain on the scale below. Circle the number that represents your assessment of A’s pain. | | | | |
|  | 0 1 2 3 4 5 6 7 8 9 10 | | |  |
| No pain/discomfort Worst  Pain/discomfort | | | | |
| 3. Your assessment, above, is made two hours after he received morphine 2 mg intravenous (IV). Half hourly pain ratings following the injection ranged from 6 to 8 and he had no clinically significant respiratory depression, sedation, or other untoward side effects. He has identified 2/10 as an acceptable level of pain relief. His physician’s order for analgesia is “morphine IV 1–3 mg q1h (every hour) PRN (as needed) pain relief.”  Check the action you will take at this time.  1) Administer no morphine at this time.  2) Administer morphine 1 mg IV now  3) Administer morphine 2 mg IV now  4) Administer morphine 3 mg IV now | | | | |

| * **Case 2**: B is 75 years old patient as having back pain. As you enter his room, she is lying quietly in bed and grimaces as she turns in bed. Your assessment reveals the following information: BP, 120/80 mm Hg; HR, 80 beats/min; R, 18 beats/min; on a scale of 0 to 10 (0, no pain/discomfort; 10, worst pain/discomfort) he rates his pain as 8. | | | | |
| --- | --- | --- | --- | --- |
| 4. Circle the number that you rate the B’s pain. | | | | |
|  | | 0 1 2 3 4 5 6 7 8 9 10 |  | |
| No pain/discomfort Worst  Pain/discomfort | | | | |
| 5. On the patient’s record you must mark his pain on the scale below. Circle the number that represents your assessment of B’s pain. | | | | |
|  | 0 1 2 3 4 5 6 7 8 9 10 | | |  |
| No pain/discomfort Worst  Pain/discomfort | | | | |
| 6. Your assessment, above, is made two hours after she received morphine 2 mg IV. Half hourly pain ratings following the injection ranged from 6 to 8 and she had no clinically significant respiratory depression, sedation, or other untoward side effects. She has identified 2/10 as an acceptable level of pain relief. Her physician’s order for analgesia is “morphine IV 13 mg q1h PRN pain relief.”  Check the action you will take at this time:  1) Administer no morphine at this time.  2) Administer morphine 1 mg IV now  3) Administer morphine 2 mg IV now  4) Administer morphine 3 mg IV now | | | | |
